# Forme pseudotumorale de tuberculose oculaire: à propos de 2 cas

**DOI:** 10.11604/pamj.2020.36.147.20571

**Published:** 2020-07-02

**Authors:** Incaf Elboukhani, Asmaa Siati, Issam Errachiq, Adil Mchachi, Leila Benhmidoune, Rayad Rachid, Mohamed Elbelhadji

**Affiliations:** 1 Service d’Ophtalmologie Adulte, Hôpital 20 Août 1953, CHU Ibn rochd, Université Hassan II de Casablanca, Maroc

**Keywords:** Tuberculose oculaire, pseudotumeur, corps ciliaire, *Mycobacterium tuberculosis*, traitement antituberculeux, Ocular tuberculosis, pseudotumor, ciliary body, mycobacterium tuberculosis, anti-TB treatment

## Abstract

Les manifestations oculaires de la tuberculose sont non spécifiques et polymorphes pouvant toucher toutes les tuniques de l’œil et entrainer une perte visuelle sévère en l’absence d’un traitement précoce et adapté. Nous rapportons 2 cas de forme pseudo tumorale de tuberculose oculaire ayant bien évolué sous traitement anti bacillaire; le premier présentant un éclatement spontané récent du globe avec issue d’une masse bourgeonnante charnue et suppurée de 10cm/6cm, le second patient présente un granulome du corps ciliaire mimant un mélanome; puis nous discutons les particularités cliniques et thérapeutiques de cette affection.

## Abstract (en)

Ocular manifestations of tuberculosis are non-specific and polymorphic as they can affect all the tunics of the eye and cause severe visual loss in the absence of early and appropriate treatment. We here report 2 casesof pseudotumoral ocular tuberculosis with favorable outcome under antibacillary treatment; the first patient recently had had spontaneous burst of the eyeball with exit of a burgeoning fleshy and suppurative mass measuring 10cm/6cm, the second patient had granuloma of the ciliary body mimicking melanoma; then we here discuss the clinical and therapeutic particularities of this disease.

## Introduction

La tuberculose est une maladie infectieuse systémique grave due au *Mycobacterium tuberculosis* [1]. C’est la première cause de mortalité et de morbidité infectieuse dans les pays en voie de développement où elle sévit à l’état endémique. L’augmentation des cas d’infection par le VIH ou syndrome d’immunodéficience acquise (SIDA) a été impliquée dans l’incidence croissante de cette affection dans les pays industrialisés [2]. Les manifestations oculaires de la tuberculose sont non spécifiques et polymorphes, réalisant divers tableaux cliniques et entrainant une perte visuelle sévère en l’absence d’un traitement précoce et adapté. Ces caractéristiques cliniques sont à l’origine de sa difficulté diagnostique [3]. Les présentations les plus courantes sont l’uvéite antérieure chronique, la choroïdite et la sclérokératite. Plus rarement, les patients peuvent initialement présenter des symptômes oculaires simulant une tumeur maligne intra-oculaire. Nous rapportons 2 cas de formes cliniques atypiques de granulome pseudo-tumoral intra-oculaire, mimant une tumeur maligne, au cours d’une atteinte systémique tuberculeuse, dont nous décrivons les particularités cliniques, radiologiques, thérapeutiques et évolutives; la trithérapie antibacillaire ayant entraîné une régression spectaculaire des lésions oculaires et systémiques avec récupération visuelle partielle pour un cas.

## Patient et observation

**Figure 1:**
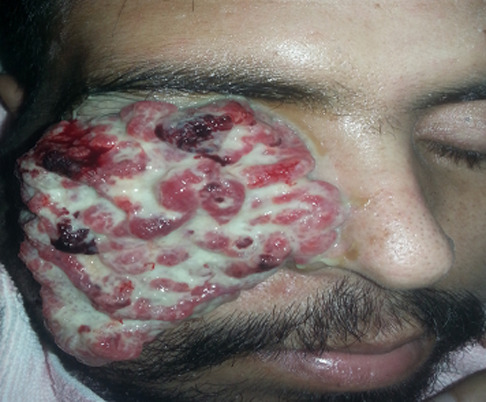
masse bourgeonnante et suppurée de 10cm/6cm

**Figure 2:**
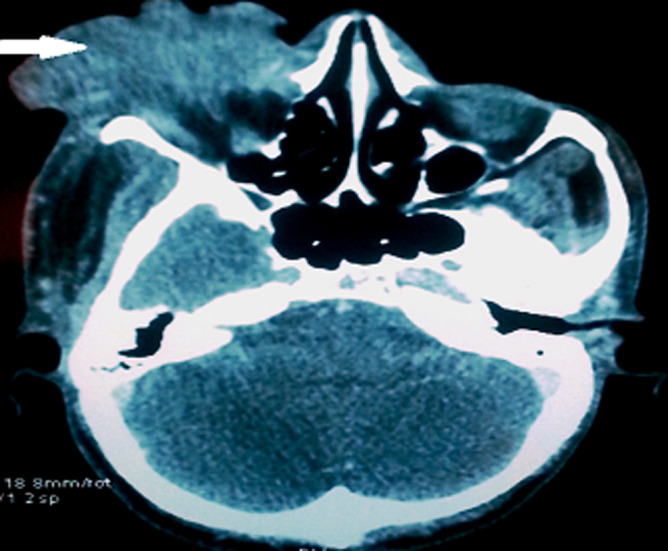
processus ulcéro-bourgeonnant endo-oculaire avec infiltration des muscles oculo-moteurs sans lyse osseuse, sans lyse osseuse et infiltration de la graisse intra et extra-conique

**Figure 3:**
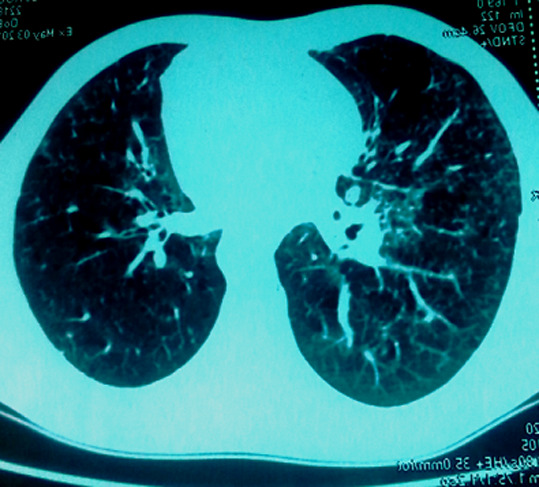
TDM thoracique: multiples nodules pulmonaires avec adénopathies mediastinales

**Figure 4:**
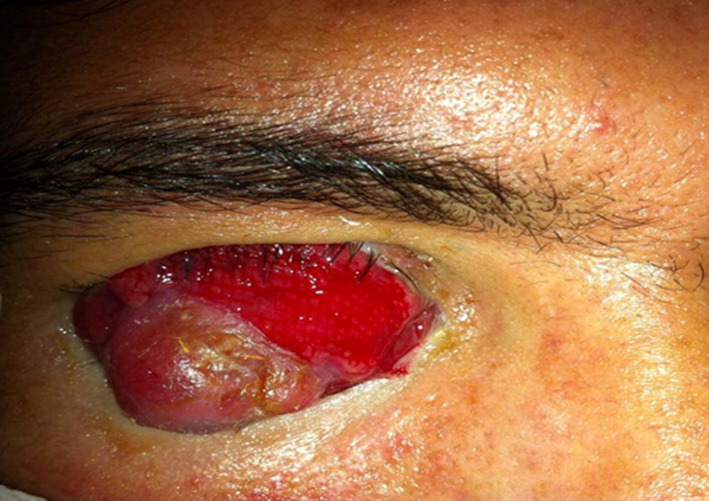
après traitement anti-tuberculeux, avec recul de cinq mois, nette régression de la taille de la masse bourgeonnante

**Figure 5:**
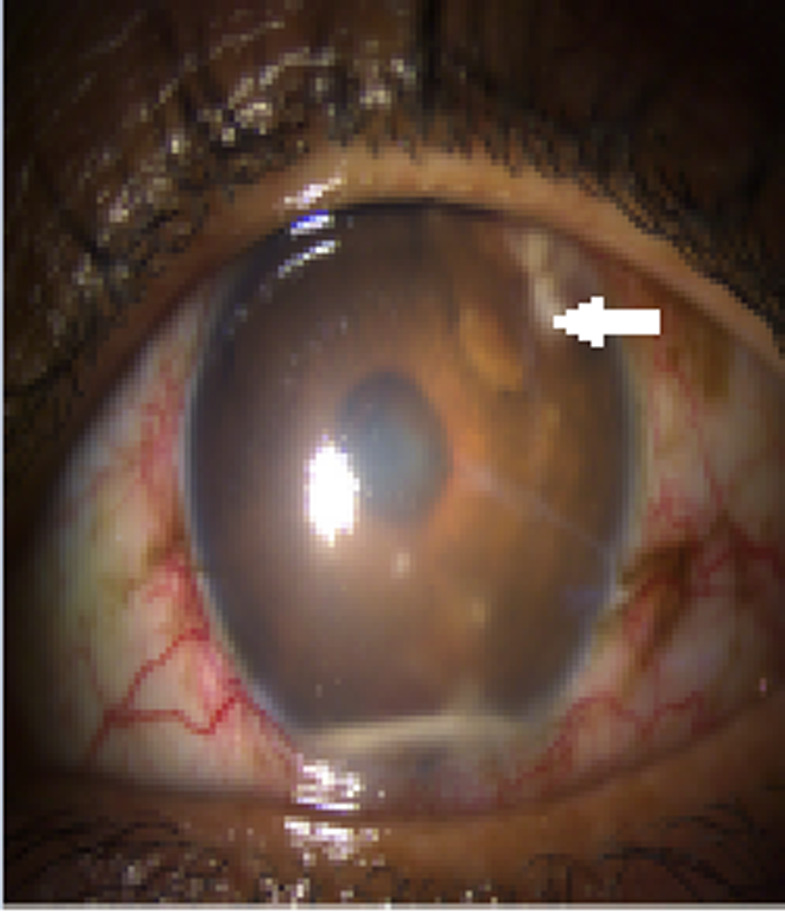
uvéite antérieure avec bombement irien supero-temporal

**Figure 6:**
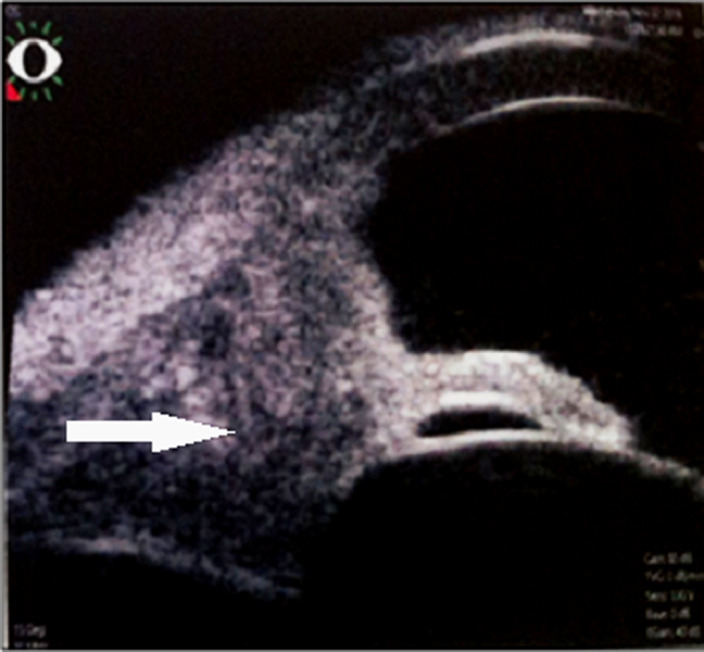
UBM montrant un aspect en faveur d’un processus tumoral du corps ciliaire

**Figure 7:**
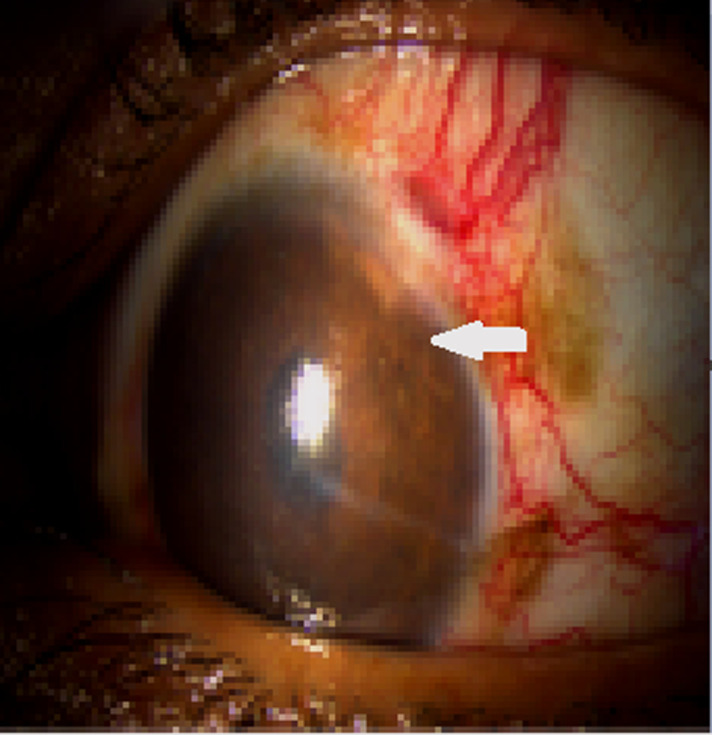
régression du bombement irien après un mois de traitement anti-bacillaire

Le premier cas est celui d’un patient âgé de 29 ans, sans antécédents pathologiques ni notion de comptage tuberculeux, consultant pour une exophtalmie douloureuse droite évoluant depuis un an avec éclatement spontané récent du globe, l’examen ophtalmologique de l’œil droit concerné objective un éclatement du globe oculaire, avec issue d’une masse bourgeonnante charnue et suppurée de 10cm/6cm (Figure 1), l’examen de l’œil gauche retrouve une acuité visuelle à 10/10^e^avec un segment antérieur et un fond d’œil normaux. L’examen général objective un adénophlegmon latéro-cervical gauche. L’IRM crânio-orbitaire met en évidence un volumineux processus tumoral ulcèro-bourgeonnant endo-oculaire mesurant 9/6/4cm envahissant la glande lacrymale et les paupières avec infiltration des muscles oculo-moteurs et de la graisse intra et extra-conique sans lyse osseuse (Figure 2). L’IRM cervicale a objectivé un magma d’adénopathies cervicales et sus-claviculaires avec infiltration de la graisse masseterienne. Une biopsie du processus oculaire bourgeonnant a été réalisée concluant à une inflammation granulomateuse tuberculoïde, avec nécrose caséeuse compatible avec une tuberculose évolutive. La TDM thoracique objective de multiples nodules pulmonaires avec adénopathies médiastinales (Figure 3). La recherche de BK dans les crachats est revenue négative, la bronchoscopie pulmonaire a retrouvé 3 masses bourgeonnantes bronchiques nécrosées dont l’examen anatomo-pathologique révèle un granulome tuberculoïde sans nécrose caséeuse. Un traitement antibacillaire a été prescrit pour une durée de 9 mois selon le protocole: rifampicine, isoniazide, pirazinamide et éthambutol pendant 2 mois, puis 7 mois de rifampicine et d’isoniazide. L’évolution a été marquée après un recul de cinq mois, par une nette régression de la taille de la masse bourgeonnante (Figure 4). Le second cas est un patient de 51ans consultant pour un œil gauche rouge, douloureux avec baisse d’acuité visuelle évoluant une semaine auparavant. L’examen ophtalmologique de l’œil concerné retrouve une acuité visuelle (AV) à 5/10, une hyperhémie conjonctivale, un œdème de cornée, tyndall +++ et fibrine en chambre antérieure (CA), avec un bombement irien supéro-temporal (Figure 5). Le reste de l’examen était gêné et l’examen de l’œil controlatéral était sans particularité. L’*ultrasound biomicroscopy*(UBM) a objectivé la présence d’une tumeur du corps ciliaire dont l’aspect est compatible avec un mélanome du corps ciliaire (Figure 6). Une ponction de la chambre antérieure avec étude cytologique a objectivé un liquide pauci-cellulaire sans signes de malignité. Un bilan général (pulmonaire et hépatique) à la recherche d’éventuelles lésions secondaires est réalisé révélant une tuberculose pulmonaire (BAAR +), le patient a été mis sous traitement anti bacillaire pour une durée de 9 mois selon le protocole: rifampicine, isoniazide, pirazinamide et éthambutol pendant 2 mois, puis 7 mois de rifampicine et d’isoniazide. L’évolution, après un recul de deux mois, fut marquée par une disparition du tyndall en CA et du bombement irien (Figure 7), l’examen de contrôle affirme la régression de la taille de la masse du corps ciliaire.

## Discussion

La tuberculose est une maladie infectieuse aiguë ou chronique causée par un bacille acido-alcoolo résistant. Le nombre de cas de tuberculose, qui avait été en baisse constante, a commencé à augmenter dans de nombreux pays industrialisés au milieu des années 1980. Avant l’ère de l’infection par le VIH, 83% des cas de tuberculose étaient limité aux poumons. Aujourd’hui, la tuberculose extrapulmonaire est plus commune. Celle-ci peut impliquer les ganglions lymphatiques (30%), la plèvre (23%), le système digestif (12%), les os (10%), les articulations (10%), et les méninges (5%). Les cas de tuberculose miliaire étendue représentent 7% des cas. La tuberculose oculaire est rare. Elle se voit surtout dans les formes miliaires, exceptionnellement dans le cadre d’une primo-infection et ceci malgré sa recrudescence avec le SIDA. Toutes les structures de l’œil peuvent être atteintes à des fréquences variables. Les localisations les plus habituelles étant au niveau de la cornée et de la conjonctive. L’atteinte choroïdienne est rare et atypique et la forme pseudotumorale est, selon la revue de la littérature, exceptionnelle [4]. Cette atteinte est reconnue pour la première fois en 1830 par Gueneau De Mussy. Mais c’est en 1867, que Coheinheim démontra que les nodules choroïdiens sont identiques aux tuberculomes des autres organes. Le diagnostic de tuberculose intra-oculaire est le plus souvent fait indirectement à partir d’une tuberculose systémique patente confirmée ou non bactériologiquement et des lésions oculaires caractéristiques [5]. Les foyers intraoculaires sont le plus souvent secondaires à une autre localisation surtout pulmonaire et/ou méningée.

Il existe diverses localisations d’un granulome tuberculeux; le tuberculome choroïdien, survenant surtout chez le sujet débilité, il peut s’étendre vers le segment antérieur, entraînant une uvéite antérieure hypertensive. En l’absence de traitement il peut entraîner une perforation du globe oculaire. Plus rarement, la scléro-uvéite tuberculeuse peut simuler une tumeur oculaire et être associée à une sclérite nécrosante avec perforation sclérale. Le tuberculome du corps ciliaire est beaucoup plus rare. Le diagnostic positif se fait sur un faisceau d’arguments de présomption: un comptage tuberculeux récent, massif surtout en pays d’endémie tuberculeuse, un terrain débilité, immunodéprimé (SIDA, post-partum, post-abortum...), une autre localisation patente notamment pulmonaire, dont l’aspect radio-clinique est très évocateur, étayé par une intradermoréaction à la tuberculine positive. Chez nos patients, immunocompétents, le diagnostic de la tuberculose oculaire a été posé par la découverte simultanée d’une localisation pulmonaire et confirmée par la bactériologie. Le diagnostic biologique repose surtout sur les résultats de l’intradermoréaction à la tuberculine et la recherche des bacilles de Koch dans les crachats. La mise en évidence du germe *Mycobacterium tuberculosis* au niveau des tissus ou des liquides intraoculaires est difficile à réaliser et peu fructueuse vue la faible concentration du germe dans l’humeur aqueuse ou le vitré. Mais elle est actuellement possible grâce à la technique de la “Polymerase Chain Reaction” [5].

Barondes [6] propose l’endobiopsie choriorétinienne qui semble une méthode intéressante mais nécessite d’être étayée par d’autres études. Le diagnostic histologique est le plus souvent réalisé sur pièce d’énucléation [7], il révèle un granulome inflammatoire caractéristique, fait de cellules épithélioïdes, gigantocellulaires avec macrophages et lymphocytes or seules les lésions riches en caséeum retrouvent ce granulome typique de tuberculose. Les tests sérologiques ne sont guère fiables. Enfin, le diagnostic positif à partir d’un essai thérapeutique de 15 jours par l’isoniazide est peu concluant d’après Holland [8] et l’utilisation de ce test est à éviter surtout devant le risque élevé de développer une résistance à l’isoniazide. Pourtant, cette méthode avait été fortement recommandée par Abrahams [9] dans certains cas particuliers, notamment les patients présentant une uvéite ou iridocyclite chronique avec intradermoréaction à la tuberculine positive. Ces deux observations, nous ont aussi posé un problème de diagnostic différentiel avec un processus tumoral oculaire. L’apport du scanner et de l’IRM orbito-encéphalique ainsi que l’atteinte pulmonaire dans un pays d’endémie ont permis de redresser le diagnostic. En effet, le scanner et l’IRM ont montré une prise de contraste et un aspect en hyposignal T1 et isosignal T2 à l’IRM. Le traitement antituberculeux instauré au Maroc (tuberculose grave), basé sur le tri ou quadrithérapie (si l’examen direct des crachats est BK positif): 2 mois de SHRZ puis 7 mois de RH, associée le plus souvent à une corticothérapie, s’avère efficace avec une durée de traitement minimale de 9 mois. Documenté par les scanners et les rétinographies successives, prouvant d’une part, une régression remarquable des lésions avec récupération fonctionnelle totale au bout de 3 mois de traitement et d’autre part, que sous traitement adapté et bien suivi, l’atteinte intra-oculaire n’est pas l’apanage des seules tuberculoses au stade terminal comme cela a été longtemps admis.

## Conclusion

La tuberculose pseudo-tumorale est très rare, Elle peut simuler des néoplasmes oculaires. La présence d’une autre atteinte systémique doit faire évoquer le diagnostic de tuberculose. L’imagerie joue un rôle fondamental pour le diagnostic lésionnel et aussi pour le suivi post-thérapeutique. Le diagnostic précoce est primordial, car la lésion est réversible sous traitement antituberculeux, évitant ainsi un traitement agressif de l’œil atteint. Un diagnostic tardif et un retard de prise en charge peuvent engager le pronostic visuel ou vital du patient.
